# A Study of Ground Deformation in the Guangzhou Urban Area with Persistent Scatterer Interferometry

**DOI:** 10.3390/s90100503

**Published:** 2009-01-15

**Authors:** Qing Zhao, Hui Lin, Liming Jiang, Fulong Chen, Shilai Cheng

**Affiliations:** Institute of Space and Earth Information Science, The Chinese University of Hong Kong, Room 615, Esther Lee Building, Shatin, N.T., Hong Kong, P.R. China

**Keywords:** Persistent Scatterer Interferometry, Ground subsidence, Guangzhou city

## Abstract

The Interferometric Point Target Analysis (IPTA) technique and Advanced Synthetic Aperture Radar (ASAR) images acquired over Hong Kong from 2007–2008 were used to detect ground deformation in the urban area of Guangzhou city in South China. A ground deformation rate map with scattered distribution of point targets shows the maximum subsidence (rise) rate as high as -26 to -20 mma^-1^ (16–21 mma^-1^), implying that the study area is an active zone for ground deformation. Based on the point target map, a contour ground deformation rate map is generated. The map shows three major subsidence zones located in the middle-west, the east, and the southwest of the study area, respectively. All the six ground collapse accidents that occurred in 2007–2008 fall within the subsidence zones, qualitatively validating the IPTA results. Ground subsidence and geological conditions on Datansha Island are examined. The results indicate that the local geological conditions, such as limestone Karst geomorphology as well as silt layers characterized by high water content, high void ratio, high compressibility, low bearing capacity and low shear strength, and underground engineering projects are responsible for ground subsidence and ground collapse accidents occurred there.

## 1. Introduction

Previous investigators have pointed out that the ground subsidence is a major geological hazard in the coastal cities in China [1, 2]. The causes of this hazard are quite complicated, but can be loosely divided into two categories: natural subsidence and the human-induced subsidence. The natural subsidence mainly includes deformation of soft soil, and Karst geomorphologic collapse, while the human-induced subsidence results from over-pumping of underground water, land reclamation, underground mining, and concentrated construction [3–5].

Guangzhou city, the study area of this work, is a mega-city and a major industrial development center in South China. In recent years, the development of this city has severely been hampered by problems induced by ground subsidence. Investigations indicate that the main factors causing ground subsidence in Guangzhou include natural factors, such as Karst geomorphologic collapse, and deformation of soft soil, and human activities, particularly activities such as over-pumping underground water from the groundwater supply source located in the Karst geomorphologic structure and subway construction [5–7]. Thus, development of new technology for accurate and dynamic detecting of ground subsidence in the Guangzhou urban area is of significance for engineering design, city development zoning, and decision-making.

The Permanent Scatterer (PS) technique was first developed in 2000 by a research team at the Politecnico di Milano (POLIMI), Italy [8, 9]. The primary aim of this development was to overcome the limitation of temporal and geometrical de-correlation of synthetic aperture radar (SAR) interferometry, which prevents the techniques from being an operational tool in practice. Meanwhile, this new technology also overcomes the limitation of atmospheric effects, which can greatly degrade the accuracy of the interferometric results [8, 9]. The spatial density of PS is much higher than the grids that are generated by the traditional point-based methods, such as leveling surveying and Global Positioning System (GPS) technology. In particular, in the urban areas the PS density may reach as high as 100 PS km^-2^. The accuracy of deformation measurements may be raised to 0.1–1 mma^-1^ on the averaged line-of-sight deformation rate, and 1–3.5 mm on a single measurement [9, 10]. This implies that the PS technique can be used as an operational tool to measure ground deformation with a high accuracy and a high spatial PS grid density. Meanwhile, a number of modified techniques are developed to further improve the PS technique, such as Coherent Target Monitoring (CTM), Interferometric Point Target Analysis (IPTA), Small Baseline Subset Approach (SBAS), Spatial Temporal Unwrapping Network algorithm (STUN), and Stanford Method for PS (StaMPS) named Persistent Scatterer Interferometry (PSI) [11]. Recently, Stramondo *et al.* used differential SAR interferometry (DinSAR) to detect ground deformation after the Sichuan earthquake [12].

In this paper, the Interferometric Point Target Analysis (IPTA) technique, a PSI technique, is used to investigate ground deformation in the urban areas of Guangzhou city, which is located at the Pearl River Delta in South China. A time series of ENVISAT (European Space Agency satellite) ASAR images, taken from 19 March 2007 to 12 May 2008, are used to generate ground deformation fields and interpretation maps in the study area. The derived ground subsidence data are then compared with reported ground observation results and analyzed in detail. The mechanisms for causing the ground subsidence in Guangzhou urban area are also analyzed. This work is also a demonstration study for evaluating the general performance of the applied PSI technique in detecting ground subsidence with limited SAR images.

## 2. Methodology

### 2.1. Study area

Guangzhou city, the study area of this work, is the capital of Guangdong province in South China. The land area of Guangzhou city is 7,434.4 km^2^ and the recorded population at the end of 2006 was 9,754,600, making it the third most important metropolitan area in China [13]. Guangzhou harbor is an important navigation center in South China, which is connected with the outside world through the waterways of the Pearl River and the South China Sea. Guangzhou is the economic center of South China. In 2007, its GDP reached US $92 billion, ranking Guangzhou 6th among the Chinese cities [13]. Thus, thousands of construction projects are currently underway or in the planning stages.

**Figure 1.**
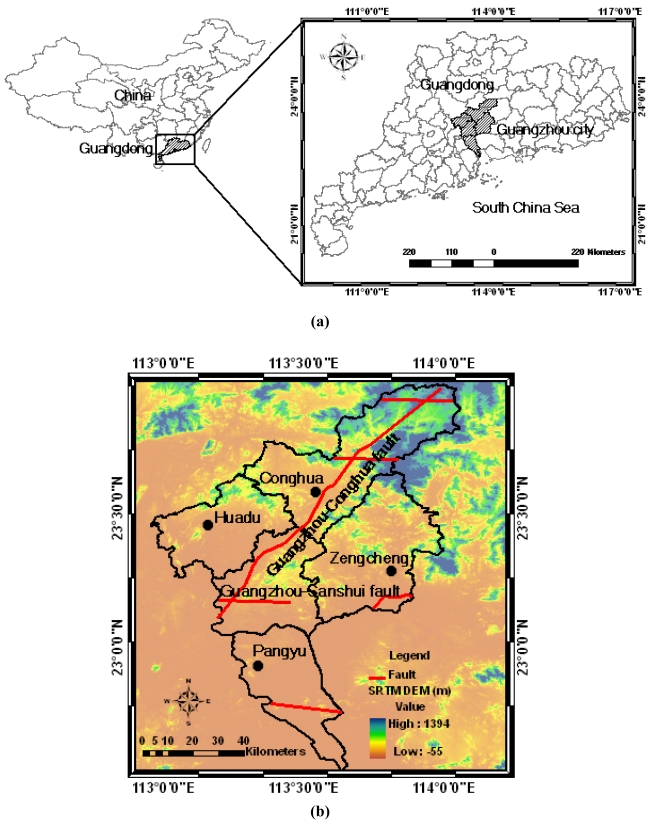
(a) Location of study area in Guangdong Province. (b) Map of study area. Active faults are marked in red (partially referred to [5, 13]).

Geographically, Guangzhou city covers an area from 112°57′ to 114°3′E and from 22°26′ to 23°56′N (see Figure 1). This region is located at the northern margin of the Pearl River Delta. Guangzhou city is within a humid subtropical climate zone controlled by the Asian monsoon, which is characterized by wet summers with high temperatures and high humidity and cool dry winters. The mean annual temperature is 22.8°C. The average annual precipitation can reach 1,982.7 mm, concentrated in the flood season from April to October with frequently occurring typhoon and rainstorm landfalls. Generally, in the wet season, the underground water table rises and the flow rate increases. Dissolution becomes stronger. The collapse of Karst-cave roofs is common. After the wet season, the underground water table decreases. The Karst-cave roofs can also collapse due to the effects of gravity. Thus, the high annual precipitation rate and uneven annual precipitation distribution constitute a significant mechanism for ground collapse in the Karst geomorphologic areas [5].

The landform of Guangzhou city corresponds to hilly country, with altitudes of about 200–1,000 m. The north terrain is higher than the south [5]. The urban area of Guangzhou is located in the Guangdong Depression Belt of the South China fold system. There are more than 40 faults and 30 folds. The Guangzhou-Sanshui fault and Guangzhou-Conghua fault divide the city into three sub-geological structure units: Guangzhou-Huadu fault basin, Zengcheng salient, and the Pearl River Delta Depression [5]. These large-scale structures constitute potential causes of ground surface deformation.

The northern and western portions of Guangzhou city, belonging to Guangzhou-Huadu fault basin, are rich in huge limestone. Thus, the limestone corrosion fractures and the Karst caves are gestated there. The depth of silt layer in the Pearl River Delta reaches about 15–40 m. This kind of soft soil, with high compressibility, lower permeability and bearing capacity, easily leads to ground deformation damage, thixotropic flow, foundation sinking, sand liquefaction, and seismic subsidence [14–16].

### 2.2. ASAR data and data pre-processing

The satellite SAR images taken by ENVISAT satellite constitute a baseline for this work. ENVISAT satellite is an advanced polar-orbiting earth observation satellite launched by the European Space Agency in March 2002. The ASAR instrument on board the ENVISAT operating at C-band ensures continuity with the image mode (SAR) and the wave mode of the ERS-1/2 Active Microwave Instrument (AMI). It features enhanced capability in terms of coverage, range of incidence angles, polarization and modes of operation [17].

All the ASAR images used in this work were acquired at the Hong Kong Remote Sensing Ground Receiving Station, which was established at the Chinese University of Hong Kong in 2006. The antenna of the receiving station covers a circular area with a radius of over 2,500 km centered in Hong Kong. Our study area is near Hong Kong, so that it is located near the center of circular coverage of the antenna. This work uses 10 images of Image Mode raw data taken from March 19, 2007 to May 12, 2008 as listed in Table 1, which have been pre-processed at the receiving station before delivering to the users. Images taken between December 24, 2007 and May 12, 2008 are not included in our study, since they were acquired with alternative polarization mode.

The images listed in Table 1 are single look complex (SLC) image products generated from raw ASAR data, which are geometrically corrected. The images have also been radiometrically calibrated using Gamma software [18].

### 2.3. IPTA technique

As mentioned in Introduction, the concept of the Permanent Scatterer (PS) technique was first raised by a research team at POLIMI [8, 9]. The motivation for developing this technique is to overcome the limitations of temporal and geometrical de-correlation, which prevents SAR interferometry from being an operational tool in practice, and atmospheric effect, which can greatly degrade the accuracy of the interferometric results [8, 9]. The PS technique identifies the so-called permanent scatterers (PSs), which are image pixels coherent over long time series. The coherent values may be quite high even for interferogram baselines that are longer than the critical length. Thus, the technique may exploit the interferometric phase and attain millimetric ground settlements based on the sparsely distributed PSs. The PS technique fully exploits a great number of archived SAR images, even the interferometric SAR images with baselines larger than the so-called critical baseline.

In this study, the Interferometric Point Target Analysis (IPTA) technique and ASAR images are used to generate ground deformation maps of Guangzhou urban area. The IPTA is one of PSI techniques [19, 20]. The idea of IPTA technique is to use point targets for completely exploiting achieved data even for the interferometric pairs with long baselines. Thus, the technique is capable of reducing the errors resulting from the atmospheric path delay and increasing the temporal sampling rate. The selection criteria of point targets are mainly based on low spectral diversity, high backscattering intensity and low temporal variability of the SLC intensity. Another reason for this study choosing IPTA technique is that there is commercial software available. Our quite long term experience to use this software indicates that it is quite efficient and user-friendly.

The unwrapped interferometric phase *ϕ_unw_* model of IPTA is the sum of topographic *ϕ_topo_*, ground deformation *ϕ_def_*, differential path delay phase *ϕ_atm_*, and phase noise *ϕ_noise_* terms [20]:

(1) $$\phi_{\text{unw}}=\phi_{\text{topo}}+\phi_{\text{def}}+\phi_{\text{atm}}+\phi_{\text{noise}}$$

Across the layers of data stack, it is considered that the phase model is a linear dependence of the topographic phase on the perpendicular baseline component. A linear regression, which depends on the perpendicular baseline component, is employed. The slope of the regression line represents a relative height correction value. Consequently, the two-dimensional (2D) regression analysis, which is based on the perpendicular baseline and the time and treating deformation rate as a constant, is performed only for pairs with short spatial perpendicular baseline taken at all point targets. Next, for large stacks, a non-linear regression is performed using the wrapped phase. However, spatial phase unwrapping is prior to the non-linear regression for small stacks. The residual phase includes the atmospheric delay phase, non-linear deformation and an error term. According to different spatial and temporal dependences among different phase terms, an approach of a step-wise, iterative improvement of different parameters is used to separate different parameters and to refine the phase model expressed in Equation (1). By extending the point list to the refined phase model, there is an expectation that a few point targets may also be found in non-urban areas, enlarging the spatial coverage of point targets.

In summary, the IPTA technique is characterized by the following features: 1) It exploits the temporal and spatial characteristics of interferometric signatures collected from point targets [20]; 2) There are two different approaches, which are used to select point target candidates. For large data stacks, the criteria for an initial selection of point target candidates include low temporal variability of the backscattering coefficient. In the case of small data stacks, the temporal variability criteria to select point target candidates become less reliable for statistical reasons. Thus, for small data stacks, the criteria are high backscattering and low spectral phase diversity. Combining the two approaches for limited data stacks may increase the reliability and confidence level of point target candidates [18]. Both the two approaches for selection point target candidates are used in our experiments.

### 2.4 Field observed ground deformation

**Figure 2.**
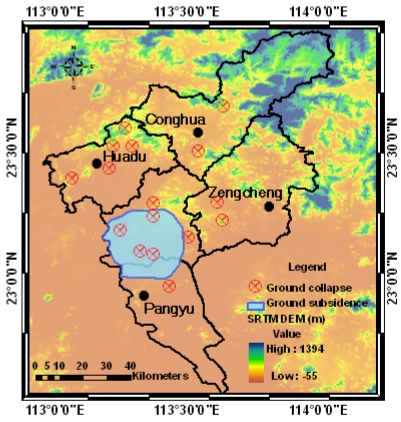
Distribution of ground collapse and ground subsidence in Guangzhou City (partially referred to [5]).

Previous investigations indicated that in Guangzhou area ground subsidence and ground collapse may occur anytime throughout the year. The frequency has steadily increased continuously in recent years, mainly due to over-pumping of underground water to satisfy the need for drinking water and water for industrial uses. The peak phase of occurrence frequency of serious events lasts from April to August. In this local spring-summer rainy season, the groundwater level rises significantly. It becomes the main factor causing covered Karst collapse. The regional distribution of ground collapse and ground subsidence in Guangzhou city is shown in Figure 2. One can see that ground subsidence is mainly distributed in the urban area of Guangzhou city and that ground collapse mainly occurs in the Guangzhou-Huadu basin.

**Figure 3.**
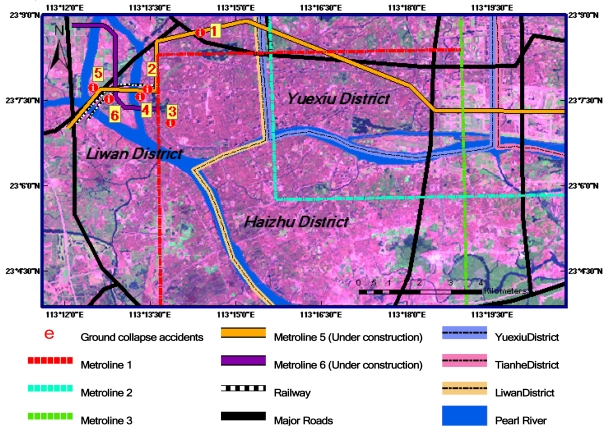
The spatial distribution of ground collapse accidents in 2007–2008 are coded from 1 to 6. The underground metro-lines and district boundary lines are also marked. (The background image is Landsat TM false color composite image. The spatial resolution is 30m.).

The spatial distribution of main ground collapse accidents occurring from 2007 to 2008 is shown in Figure 3. These accidents are mainly distributed along the underground metro-lines around Datansha Island in Liwan District in the southwest of Guangzhou city. The related information of six ground collapse events coded in Figure 3 is listed in Table 2. Fortunately, the occurrence dates of the accidents were all in the ASAR image receiving time scope (see Table 1). This provides a useful opportunity to validate the ground deformation data derived from ASAR images.

## 3. Mapping of ASAR-derived ground deformation

**Figure 4.**
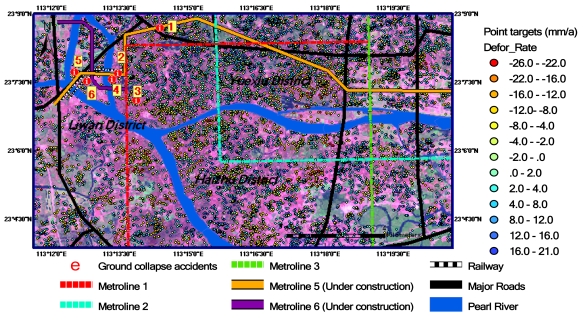
A ground deformation rate map of IPTA-derived point targets in Guangzhou using ASAR data listed in Table 1. Deformation rates are color-coded, as shown on the right-hand side. Numbers 1–6 as marked in Figure 3 show the locations of ground collapse accidents that occurred in 2007–2008. (The background image is Landsat TM false color composite image. The spatial resolution is 30 m.).

Using the IPTA technique and ASAR data listed in Table 1, a ground deformation rate map of the Guangzhou urban area is generated, as shown in Figure 4. One can see that the data points are unevenly but densely distributed over the study area. A total number of 21,265 point targets are detected. The average density is 150 points km^-2^. The maximum subsidence rate is -26 to -20 mma^-1^, and the maximum rise rate is 16–21 mma^-1^. These values are much larger than the normal ground deformation rates induced solely by crustal movements. Gumen and Kissin [21] measured an average vertical amplitude of crustal movements in Byelorussia as 4 ± 3 mma^-1^ ranging from 0 to 10 mma^-1^ from 1975 to 1986. Mizouo [22] gave a vertical amplitude of earth crustal movements in Japan as 1–4 mma^-1^ for the wavelength of 20–100 km (a scale of our study area). Therefore, 5 mma^-1^ should be a reasonable estimate for the normal ground deformation rates induced solely by crustal movements. This indicates that the ground deformation rates in the Guangzhou urban area derived from this study are 4–5 times greater than the normal ground deformation rates induced solely by crustal movements, implying that the study area is an extremely active area for ground deformation.

**Figure 5.**
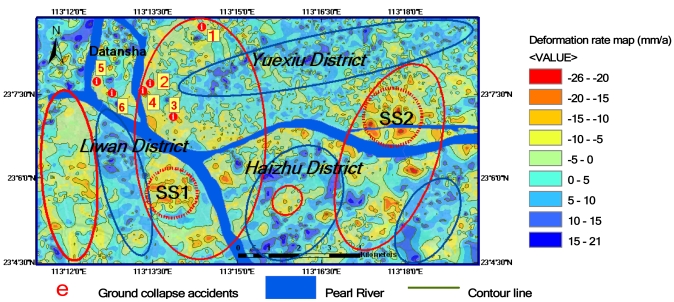
A contour map of ground deformation in Guangzhou city.

In order to further examine the spatial distribution features of ground deformation in the study area, a contour map is generated using the data in Figure 4 and interpolation technology. The results are shown in Figure 5. From this ground deformation contour map, one can see that all the sites of 6 ground collapse accidents that occurred in 2007–2008, as listed in Table 2, fall within the subsidence areas. The corresponding subsidence rates derived from ASAR images are also listed in Table 2 as a reference. One can see that all the subsidence rates at these 6 sites exceed -10 mma^-1^, and the maximum value is -20 mma^-1^. This close correlation relationship indicates that the ASAR-derived ground deformation rates are reasonable, and may be used to reveal the macro-scale features of ground deformation in the study area.

The horizontal resolution of the ground deformation rate contour map is 100 m and the vertical resolution is 2 mma^-1^. Thus, the map contains rich information on ground deformation, which is important for various users. In order to describe 2-D spatial distribution features of ground deformation in the study area, the connected subsidence (rise) areas are circled by red (blue) ellipses. One can see three major subsidence zones. The largest zone is located in the middle west of the study area, which runs from the north border to the south border and crosses the Pearl River. A high subsidence sub-region with a subsidence rate of -26 mma^-1^ is located in the south of the Pearl River as coded SS1 in the figure. Its area is about 2 km^2^. The secondary subsidence zone is located in the east of the study area. Its coverage is a little smaller than the largest one, and also distributed crossing the Pearl River. Within this subsidence zone, there is also a high subsidence sub-region with a subsidence rate of -26 mma^-1^, which is located in the north of the Pearl River as coded SS2 in the figure. Its area is about 6 km^2^. The third subsidence zone is located in the southwest of the study area. Between two subsidence zones, there is always a rise zone with a maximum rise rate of 21 mma^-1^. In the other words, the subsidence zones and rise zones are alternately distributed. The horizontal scale of the subsidence - rise patterns is about 5 km. These interesting patterns seem to show that they may be caused from the movement of local geological structure. Of cause, to conclude validation of this hypothesis, however, requires further evidence and examination.

## 4. A Case Study: Ground subsidence and geological conditions along metro-lines in Datansha Island

**Figure 6.**
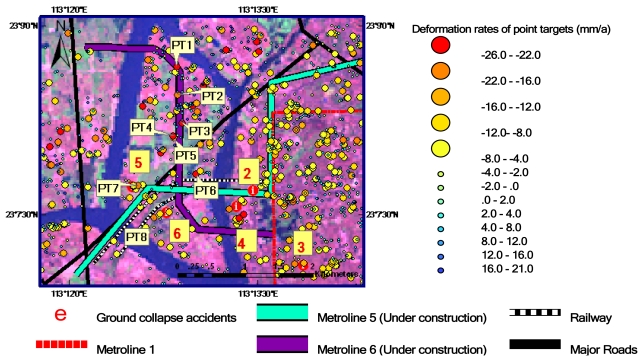
An ASAR-derived ground deformation map in Datansha Island area. PT1–PT8 are test sites along metro-lines 5 and 6. Numbers 2–6 are sites of ground collapse accidents occurred in 2007–2008.

**Figure 7.**
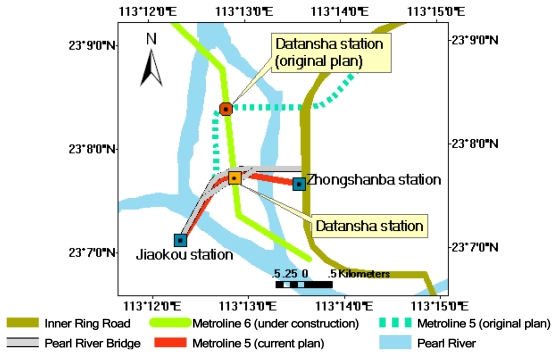
Datansha Island area and underground metro-lines (partially referred to [23]).

Two underground metro-lines under construction in Guangzhou City, Line 5 and Line 6, pass through and intersect in Datansha Island. The island is located in the center of the Pearl River in the northwest of study area as shown in Figure 6. Thus, the island is used as a test site for detecting ground subsidence caused from natural processes and underground engineering projects. Eight point targets distributed along underground metro-lines 5 and 6 in Datansha Island as shown in Figure 6 are used for this purpose. The corresponding ground subsidence rates derived from ASAR images are listed in Table 3. One can see that the mean subsidence rate reaches -19.6 ± 3.1 mma^-1^, ranging from -13.7 to -23.1 mma^-1^. These values are 2.7–4.6 times higher than the normal ground deformation rate, ± 5 mma^-1^. Moreover, all ground collapse accidents that occurred in 2007–2008 were also distributed along underground metro-lines 1, 5, and 6. These facts indicate that Datansha Island area is an active ground subsidence zone. In order to analyze the causes of ground subsidence, the test site is divided into 3 sections according to the geological conditions: section 1 from Jiaokou station to Datansha station, section 2 from Datansha station to Zhongshanba station, and section 3 is the Datansha Island segment of metro-line 6 as shown in Figure 7. Note that underground metro-lines 5 and 6 cross each other at Datansha station.

### 1) Section 1: from Jiaokou station to Datansha station

Datansha Island is a relatively flat and wide sand bank. This segment of underground metro-line 5 crosses the south portion of the island as shown in Figure 7. In the east of metro-line 5, there is the Inner Ring Road, and the Pearl River Bridge is running over it. From Figure 6, one can see that ground collapse accident 5 occurred within this section. At point target 7, near ground collapse 5, the subsidence rate is -17.3 mma^-1^.

In the feasibility study phase of the underground metro-line project, the original design of metro-line 5 was expected to use the underground line (shown as dashed line in Figure 7). Meanwhile, an open surface method was chosen to construct Datansha station. A shield method was designed to construct the section from Jiaokou station to Datansha station. However, the initial plan was soon abandoned due to the complicated engineering geological conditions. According to the original design, the Guangzhou-Sanshui fault and metro-line 5 would intersect under the western branch of the Pearl River. Fault breccia is found at Datansha station. Quaternary sandstone is also developed here. The sandstone has strong permeability and the sandstone layer also has direct hydraulic connection with the Pearl River. The bedrock is constituted by cretaceous red sandstone and carboniferous Huanglung limestone. Thus, lithology is complicated. Moreover, Huanglung limestone is well developed here. During the drilling process, a moderately large cave was found, and ground collapse occurred. Thus, all the underground lines west-eastward going through Datansha Island would inevitably pass through limestone. Therefore, the final design of this segment is to build an elevated bridge instead of underground tunnels [23].

### 2) Section 2: from Dantansha station to Zhongshanba station

From Figures 6 and 7, one can see that ground collapse accidents 2 and 4 occurred within this section. The underground tunnel for metro-line 5 from Datansha station to Zhongshanba station passes through mucky sandstone. The geological condition of some segments is silt layer and moderate coarse sandstone. The bottom of the tunnel passes through sedentary rock and strata. The shortest distance from the roof of tunnel to the bottom of Pearl River is about 5 m. The average water content of silt layer is 63.90%. Void ratio is 1.71. The average compressibility is 1.42 MPa^-1^[23]. Thus, the silt layer is characterized by high water content, high void ratio, high compressibility, low bearing capacity, and low shear strength. The mucky sandstone and moderate coarse sandstone are highly permeable and also have direct hydraulic connection with the Pearl River.

### 3) Section 3: Datansha Island segment of metro-line 6

From Figures 6 and 7, one can see that ground collapse accident 6 occurred at this segment. At point target 8, near accident 6, the ground subsidence rate reaches -17.9 mma^-1^. Metro-line 6 is designed to pass through Datansha Island from north to south.

The geological condition of Datansha Island is that carboniferous Huanglung strata are mainly distributed there. Limestone Karst caves have developed. Previous investigations showed that the Karst caves are distributed as moniliforme. The maximum height of a single cave reaches up to 21 m. According to the results of the pumping test, Huanglung limestone corrosion fissure and the Karst caves are well developed there. The water permeability coefficient of carbonates corrosion fissure and Karst caves is 112.2–135.5 md^-1^. The maximum prediction value of water inflow is 608.8–814.7 m^3^d^-1^ [24].

## 5. Conclusions and Discussion

This study uses IPTA technique and ASAR images acquired in Hong Kong to detect ground deformation in the urban area of Guangzhou city, an important industrial center in South China, which is negatively affected by ground subsidence. The major results are summarized as follows:

1. A ground deformation rate map of Guangzhou urban area with scattered distribution of point targets is generated with ASAR images acquired in 2007–2008. The maximum subsidence (rise) rate reaches up to -26 to -20 mma^-1^ (16–21 mma^-1^). These values are 3–5 times larger than the normal ground deformation rate induced solely by crustal movement, implying that the study area is an active zone for ground deformation. This agrees with the results obtained by previous investigations.
2. Based on the point target map, a contour ground deformation rate map of Guangzhou urban area is generated by an interpolation method. The horizontal (vertical) resolution of the contour map is ∼ 100 m (2 mma^-1^). The contour map shows three major subsidence zones located in the middle-west, the east, and the southwest of the study area, respectively. The maximum subsidence rate reaches -26 mma^-1^. Between two subsidence zones, there is always a rise zone with a maximum rise rate up to 21 mma^-1^. The horizontal scale of the subsidence - rise patterns is about 5 km. All the sites of six ground collapse accidents that occurred in 2007–2008 fall within the subsidence areas, qualitatively validating the IPTA results.
3. Ground subsidence and geological conditions along metro-lines in Datansha Island are examined as a case study. The analysis indicates that the local geological conditions, such as limestone Karst geomorphology, as well as silt layers characterized by high water content, high void ratio, high compressibility, low bearing capacity and low shear strength, are natural causes for ground subsidence and ground collapse accidents occurring there. Human activities in the form of underground engineering projects constitute another important cause.

This study gives the quantitative data of ground deformation in the Guangzhou urban area. It is necessary to have a precision assessment for these results and for the technique used. Although there are not simultaneous leveling data available for a point-to-point comparison with IPTA ground deformation measurements, the results of case study of Datansha Island indicate that the IPTA-derived ground subsidence rates qualitatively coincide with field observations of ground collapse events. On the other hand, in a parallel research project, the IPTA technique is applied for monitoring ground stability in Hong Kong International Airport (HKIA). For that project, there are simultaneous or near-simultaneous leveling data available for comparison with IPTA/ASAR-derived ground deformation data. Statistical analysis gives a systematical bias of 2 mma^-1^ between the two datasets (IPTA/ASAR method underestimated) with the annual deformation rates ranging from 0.1 to 10.2 mma^-1^. The correlation coefficient (R^2^) between the two datasets is 0.736 [unpublished data]. These results may serve as a close estimate for a potential precision of IPTA technique used for this study.

How many SAR images are really needed for employing IPTA technique? This is another important question. Theoretically, the more, the better, but there is not a hard threshold. Ferretti *et al.* supposed that if the quantity of SAR images is more than 30, the time series of the amplitude values of each pixel can be analyzed for detecting stable scatterers [9]. They used 41 SAR images to estimate ground subsidence in Pomona [8]. For our HKIA project, 15 ASAR images are used to generate ground deformation fields and interpretation maps. As mentioned above, the systematic bias between the field measured and IPTA/ASAR-derived ground deformation rates reaches 2 mma^-1^. We may say that although this precision is not perfect for all the users, the data are still useful at least for long term trend monitoring. For this project, serving as a demonstration study, only 10 ASAR images with a temporal coverage of one year are available for the use to generate ground deformation fields in the study area. The results indicate that important information on locations of major ground collapse disasters and horizontal distribution features of ground deformation can be extracted from the IPTA/ASAR-derived ground deformation data, demonstrating that the IPTA technique with limited archived SAR data may serve as an efficient tool for monitoring the ground deformation. The availabilities of point-to-point comparison data for the correlation/validation analysis of IPTA/ASAR-derived ground deformation data with field measurements and more SAR images would always be a hope for the future study.

The contour ground deformation rate map of Guangzhou urban area shows alternately distributed patterns of subsidence and rise zones with a horizontal scale of about 5 km. The patterns seem to indicate that they may be caused by the movement of local geological structure. To confirm this point, further evidence and analysis are needed.

## Figures and Tables

**Table 1.** Image Mode ASAR Data used in this study.

**No.**	**Acquisition Date** **(yyyymmdd)**	**Orbit No.**
1	20070319	26403
2	20070422	26904
3	20070528	27405
4	20070702	27906
5	20070806	28407
6	20070910	28908
7	20071015	29409
8	20071119	29910
9	20071224	30411
10	20080512	32415

**Table 2.** Ground collapse accidents in Guangzhou in 2007–2008 are coded from 1 to 6 in Figure 3.

**Code**	**Occurrence time**	**Location**	**Scope, x×y×z**	DR^*^mma^-1^
1	Feb 29, 2008	Huanshixi Road, Liwan	3 m × 1.5 m × 3 m	-15 to -10
2	Jan 31, 2008	Zhongshanba Road	small scope, 30 cm deep	-15 to -10
3	Oct 06, 2007	Duobao Road	300 m^2^, ∼ 6m deep	-20 to -15
4	Feb 18, 2008	Juncture of Zhongshanba Rd. Nanan Rd and Huangsha Av, Liwan	100 m^2^, deepest site over 40m	-20 to -15
	Jan 23–24, 2008	Datansha Island	200-300 m^2^, >3m deep	-15 to -10
6	Feb 02, 2008	Datansha Island	Observable	-15 to -10

* ASAR-derived ground deformation rate (vertical rate)

**Table 3.** Point targets in Datansha Island.

Metro-line	Point Target	Deformation Rate (mma^-1^)
6	PT1	-22.6
6	PT2	-18.2
6	PT3	-23.1
6	PT4	-22.2
6	PT5	-13.7
5	PT6	-21.4
5	PT7	-17.3
5	PT8	-17.9
	Mean	-19.6 ± 3.1
